# Amplified parallel antigen rapid test for point-of-care salivary detection of SARS-CoV-2 with improved sensitivity

**DOI:** 10.1007/s00604-021-05113-4

**Published:** 2021-12-06

**Authors:** Danny Jian Hang Tng, Bryan Chu Yang Yin, Jing Cao, Kwan Ki Karrie Ko, Kenneth Choon Meng Goh, Delia Xue Wen Chua, Yong Zhang, Melvin Lee Kiang Chua, Jenny Guek Hong Low, Eng Eong Ooi, Khee Chee Soo

**Affiliations:** 1 Department of Infectious Diseases, Singapore General Hospital, 20 College Road, Singapore, 169856 Singapore; 2 Programme in Emerging Infectious Diseases, Duke-NUS Medical School, 8 College Road, Singapore, 169857 Singapore; 3 Department of Head and Neck and Thoracic Cancers, Division of Radiation Oncology, National Cancer Centre Singapore, 11 Hospital Crescent, Singapore, 169610 Singapore; 4 Department of Biomedical Engineering, National University Singapore, 4 Engineering Drive 3, Engineering Block 4, Singapore, 117583 Singapore; 5 State Key Laboratory for Oncogenes and Related Genes, School of Biomedical Engineering and Institute of Medical Robotics, Shanghai Jiao Tong University, Shanghai, 200030 People’s Republic of China; 6 Department of Microbiology, Singapore General Hospital, 20 College Road, Singapore, 169856 Singapore; 7 Division of Medical Sciences, National Cancer Centre Singapore, 11 Hospital Crescent, Singapore, 169610 Singapore; 8 Oncology Academic Programme, Duke-NUS Medical School, 8 College Road, Singapore, 169857 Singapore

**Keywords:** COVID-19, SARS-CoV-2, Point-of-care detection, Antigen rapid test, Parallel flow amplification, Gold nanoparticles

## Abstract

In the ongoing COVID-19 pandemic, simple, rapid, point-of-care tests not requiring trained personnel for primary care testing are essential. Saliva-based antigen rapid tests (ARTs) can fulfil this need, but these tests require overnight-fasted samples; without which independent studies have demonstrated sensitivities of only 11.7 to 23.1%. Herein, we report an Amplified Parallel ART (AP-ART) with sensitivity above 90%, even with non-fasted samples. The virus was captured multimodally, using both anti-spike protein antibodies and Angiotensin Converting Enzyme 2 (ACE2) protein. It also featured two parallel flow channels. The first contained spike protein binding gold nanoparticles which produced a visible red line upon encountering the virus. The second contained signal amplifying nanoparticles that complex with the former and amplify the signal without any linker. Compared to existing dual gold amplification techniques, a limit of detection of one order of magnitude lower was achieved (0.0064 ng·mL^–^^1^). AP-ART performance in detecting SARS-CoV-2 in saliva of COVID-19 patients was investigated using a case–control study (139 participants enrolled and 162 saliva samples tested). Unlike commercially available ARTs, the sensitivity of AP-ART was maintained even when non-fasting saliva was used. Compared to the gold standard reverse transcription-polymerase chain reaction testing on nasopharyngeal samples, non-fasting saliva tested on AP-ART showed a sensitivity of 97.0% (95% CI: 84.7–99.8); without amplification, the sensitivity was 72.7% (95% CI: 83.7–94.8). Thus, AP-ART has the potential to be developed for point-of-care testing, which may be particularly important in resource-limited settings, and for early diagnosis to initiate newly approved therapies to reduce COVID-19 severity.

**Graphical abstract:**

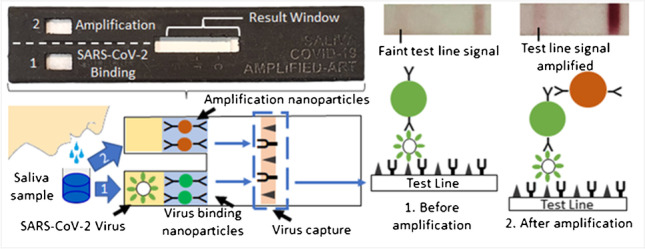

**Supplementary Information:**

The online version contains supplementary material available at 10.1007/s00604-021-05113-4.

## Introduction

Severe acute respiratory syndrome coronavirus 2 (SARS-CoV-2), the aetiological agent responsible for coronavirus disease 2019 (COVID-19), has negatively affected global health and economy [1]. Despite the unprecedented pace of vaccine deployment, demand for vaccines far outstrips supply. Low- to middle-income countries (LMICs) are most severely impacted with limited access to vaccines, leaving majority of their population vulnerable to the devastating effects of COVID-19. Furthermore, the emergence of new variants of concern such as the more transmissible delta variant is fuelling new waves of outbreaks [2]. A highly sensitive, affordable and easy-to-use test to diagnose COVID-19 rapidly for case management remains an unmet need [3]. This is especially since reverse transcription-polymerase chain reaction (RT-PCR) testing from nasopharyngeal swab samples, which is the current gold standard, would require suitably equipped laboratories and trained personnel to conduct the assay. It is also costly, with the reagent cost alone estimated at USD17.22 per test [4]. These challenges make widespread implementation of RT-PCR challenging. While urine and stool RT-PCR, which do not require labour for sample collection, had also been investigated, a meta-analysis showed that results using either sample type were inconsistent [5]. Another emerging approach is the detection of SARS-CoV-2 from exhaled breath, using RT-PCR or mass spectroscopy, with reported sensitivity ranging from 68 to 93.5% and 80 to 97.3%, respectively. However, though labour for sample collection is reduced, laboratory support is still needed for sample extraction and analyses, thus limiting their usefulness for rapid testing [6, 7].

Point-of-care tests that use easily accessible samples with high sensitivity are urgently needed. Rapid serological tests, which detect SARS-CoV-2 antibodies from a drop of blood [8], have not been proven useful for acute diagnosis, as these antibodies can remain detectable in patients who had undergone vaccination or have recovered from a SARS-CoV-2 infection for up to 13 months [9–11]. Point-of-care molecular detection techniques such as reverse transcription loop-mediated isothermal amplification (RT-LAMP), though simpler to perform than RT-PCR with adequate assay performance [12], still require skilled technologists to carry out the test [13]. On the other hand, antigen rapid tests (ARTs) can function at the point-of-care, provide results in minutes and do not require specialised equipment or labour [14]. Results are visible colorimetric signals and ARTs are also more affordable than RT-PCR, making ARTs a prime candidate to fulfil current point-of-care testing needs. Furthermore, these assays can also detect components of SARS-CoV-2 in saliva instead of nasopharyngeal swabs, which would further reduce the need for skilled healthcare personnel for sample collection [15]. Such tests could even be self-administered. A major limitation, however, of saliva-based ARTs is their low sensitivity, especially when used with non-fasted saliva samples. Fasting allows the virus to be concentrated in the saliva [16], which would be rapidly diluted after a meal or a drink. Hence, although the sensitivity of these tests on overnight-fasted saliva samples can be as high as 90%, such performance is rarely achieved in practice, as patients present throughout the day; real-world studies found sensitivities ranging from 11.7 to 23.1% [17, 18]. The requirement for overnight-fasted samples for maximal sensitivity thus limits their usefulness as point-of-care tests.

To address this, signal enhancement techniques can be used to produce a stronger colorimetric signal at lower viral loads, thus allowing the testing of unfasted saliva, while retaining sensitivity. There are 4 main methods: (i) direct test line signal enhancement, (ii) sample concentration, (iii) analyte binding nanoparticle modification, (iv) increasing nanoparticle binding time. Amongst these, direct test line signal enhancement using dual gold nanoparticle conjugate assays has shown the greatest degree of enhancement of up to 100-fold [19, 20]. These assays utilise one set of nanoparticles to bind to the analyte and a second set of nanoparticles to bind to the former nanoparticle, thereby enhancing the signal. However, the need for a linker molecule on the analyte binding nanoparticle surface to allow for the second set of nanoparticles to bind [19], leads to decreased surface area for conjugation of the analyte binding antibodies on these nanoparticles. Reduced surface area can impair analyte binding by up to fivefold [21]. Additionally, dual gold enhancement is employed in a serial format, where the analyte would encounter both sets of nanoparticles within same channel, reducing the reaction time of the analyte and the nanoparticles. Reduction in reaction time can affect analyte detection by up to twofold, resulting in sub-optimal amplification [22].

In this work, we have shown that coupling a parallel flow channel, to deliver signal amplifying, linker-free nanoparticles to a lateral flow antigen rapid test could overcome the above limitations, reproducing the high sensitivity of RT-PCR to detect SARS-CoV-2, even in non-fasted saliva samples. Additionally, to overcome SARS-CoV-2 evolution that alters the spike (S) protein structure, we designed the assay to capture SARS-CoV-2 S protein multimodally using both S-specific polyclonal antibodies and recombinant human ACE2 at the test line. A semi-quantitative mobile phone photography image processing algorithm was also applied to obtain objective measurements rather than subjective interpretation of test signal. A case–control study showed the sensitivity and specificity of our assay, relative to RT-PCR, to be 97.0% and 90.6%, respectively. These results were obtained using samples obtained an hour post-meal and thus show the ability of the AP-ART’s amplification to overcome the fasting requirement in salivary ARTs. Our findings suggest a simple, low-cost, saliva-based diagnostic suitable for use in primary healthcare settings as a point-of-care test.

## Materials and methods

### Nanoparticle conjugation

The reagents and equipment used are listed in Table S1. Covalent conjugation to create spike protein binding nanoparticles is first described. Under shaking at 800 RPM, 1.5 mg of N-(3-dimethylaminopropyl)-N-ethylcarbodiimide hydrochloride and 1.8 mg of N-hydroxysulfosuccinimide sodium salt in 50 µL of 2-(N-morpholino)ethanesulfonic acid buffer (2 g⋅l^−1^, pH 5.5) were mixed with 50 µL of gold nanoparticles and incubated for 30 min at room temperature (RT). One millilitre of phosphate buffer saline (0.01 M) and 0.05% Tween 20 solution were then added. The mixture was centrifuged at 8000 g for 10 min and the supernatant was discarded. One hundred microlitres of antibody solution (40,591-MM42, 1 mg⋅mL^−1^) was used to resuspend the pellet incubated for 4 h at RT under shaking at 800 RPM. The solution was then centrifuged at 8000 g for 10 min, the supernatant was discarded and the pellet was resuspended in 400 µL of 5% sucrose, 1% horse serum albumin (HSA) and 0.5% Tween 20. The amplification nanoparticles were prepared similarly using ab150115 antibody. Spike protein-labelled nanoparticles (S-Np) were also prepared similarly using spike protein (40,592-V05H) instead of antibody and 100 µm silica were used instead of gold nanoparticle.

### Nanoparticle characterisation

Nanoparticle concentration was determined using NanoSight and the size distribution after conjugation was determined using dynamic light scattering with the Zetasizer. Imaging of unconjugated nanoparticles was performed using transmission electron microscopy (TEM) on an FEI Tecnai G2 F20 electron microscope operating at 200 kV. The size of the unconjugated nanoparticles was determined from the TEM micrographs using manual measurement of the particle’s greatest diameter using ImageJ software and an average size of 30 particles was taken. Protein and antibody conjugation binding studies were performed by first determining the concentration of the protein or antibody for conjugation with the respective nanoparticles using the NanoDrop. Concentration was calculated using the Beer-Lambert law at 280 nm for 40,591-MM42, 40,592-V05H and ab150115 as advised by the manufacturer. For ab150115, an additional measurement was performed at 650 nm and subtracted from the absorbance at 280 nm to correct for its fluorescence emission according to manufacturer’s recommendations. After conjugation with the nanoparticles, the remaining concentration of the respective protein or antibody was again determined similarly. A control experiment was also set up where no nanoparticles were added to account for the losses in the protein or antibody during the various centrifugation and incubation steps. Protein and antibody binding was determined by subtracting the final concentration measured from the control from the final concentration after incubation with the nanoparticles.

### Amplified parallel flow antigen rapid test (AP-ART) fabrication

The protocol for the preparation of the AP-ART is adapted from standard protocol [23] and is detailed in Fig. S1 of the supplement. Briefly, using the lateral flow reagent dispenser, control lines were drawn using 200 μg⋅mL^−1^ of mouse IgG and test lines were drawn using a solution consisting of Ab01680 antibodies (1000 μg⋅mL^−1^) and ACE2 protein (100 μg⋅mL^−1^) at a rate of 0.125 mL⋅min^−1^ on nitrocellulose membrane. After drying at RT for 30 min, the membrane was blocked with 5% (w/w) HSA solution. Two sets of 2-mm wide conjugate pads were prepared: one set containing 20 µL of spike protein binding nanoparticles and another set containing 20 µL of amplification nanoparticles. Both pads were dried at RT for 2 h. The components were sequentially assembled onto an adhesive backing card in order: 5-mm wide nitrocellulose membrane, conjugation pad and absorption pad with 2-mm overlap with the membrane. A 1-mm gap was left between the first pad and second pad. All AP-ARTs were used on the day of fabrication.

## Participants and study design

A case–control study was performed and participants were tested according to the study flow shown in Fig. S2 of the supplement. All participants were tested for SARS-CoV-2 by RT-PCR on nasopharyngeal swab samples. Positive cases had SARS-CoV-2 detectable by RT-PCR with a cycle threshold of below 35 and were admitted to the Singapore General Hospital isolation ward. Controls consisted of two groups: firstly, healthcare workers (HCW) from the Singapore General Hospital who were required to undergo bi-weekly routine SARS-CoV-2 surveillance by RT-PCR testing; and secondly, patients with acute respiratory illness (ARI) admitted to the fever wards but who tested negative for SARS-CoV-2 by RT-PCR. Participants were at least 21 years of age and had not received COVID-19 vaccine. The recruitment lasted from May 2021 to Aug 2021. The study was approved by the SingHealth Centralized Institutional Review Board (application no: 2018/3045 and 2017/2387). Participants were enrolled upon written informed consent.

### Sample collection and ART testing

The analytical sensitivity of AP-ART was compared against the same lateral flow format but without amplification, using S-Np. A range of S-Np concentrations (10^5^ to 10^10^ particles⋅mL^−1^) was used. Clinical performance was evaluated using self-collected salivary samples provided by participants before breakfast without brushing their teeth and 1-h post-lunch. Patients were taught to collect their saliva via the passive drooling method. Wherever possible, longitudinal sampling was performed where samples were collected from the same participants on consecutive days. All samples were tested at the bedside within 1 h of sample collection by the study team. Saliva samples were collected within 72 h from the patient’s nasopharyngeal swab for RT-PCR testing. AP-ART testing was performed by adding 50 µL of saliva to the first channel. After 15 min, the unamplified result was recorded with a mobile phone camera. Then, 50 µL of saliva was added to the second well. After another 15 min, the amplified result was recorded similarly. As far as possible, camera settings and lighting conditions were kept the same between recordings. The images were analysed with ImageJ image processing software. Test and control line intensities were obtained by subtracting the mean intensity of each line from the mean intensity of adjacent nitrocellulose strip background. Testing using conventional ARTs (C-ART) was performed by putting 25 µL of saliva diluted with 25 µL of buffer (provided by each manufacturer) into the test well and allowed to run for the time specified by each manufacturer. RT-PCR results were not directly disclosed to study members administering the AP-ART; however, RT-PCR positivity for SARS-CoV-2 could be inferred as these participants were admitted to the isolation ward.

### RT-PCR testing

For HCWs, nasopharyngeal samples were collected as part of routine surveillance requirements and samples were sent to the Singapore General Hospital Clinical Laboratory for processing. For participants admitted to the isolation and ARI wards, nasopharyngeal samples were similarly collected and dispatched as part of their clinical care. Viral RNA isolation, purification, cDNA generation and amplification were performed according to the instructions of the manufacturer depending on the kit. The RT-PCR kits used were the Roche cubs 6800 SARS-CoV-2, Cepheid Xpert Xpress SARS-CoV-2 and Altona RealStar SARS-Cov-2. Calibration across the 3 kits was performed using a limiting dilution series of a full-genome positive control plasmid using droplet digital RT-PCR to determine copy number per microlitre of positive control plasmid. The calibration process was run with 8 replicates on separate runs by at least 3 different operators on 3 different days to ensure uniformity. Statistical analysis was performed using Probit analysis (SPSS, IBM, Armonk, NJ, USA). AP-ART results were not known to study members performing RT-PCR.

### Statistical analysis

Sample size was designed with the USFDA guidelines for development of diagnostic tests in mind, which require at least 30 positive and negative samples. Absolute test and control line intensities from testing were determined from the recorded images and processed using ImageJ. Normalised test line intensities were calculated by expressing its absolute intensity as a percentage of the absolute control line intensity. Test line intensity means before and after amplification were also compared against one another and against the control using the student’s *T* test at 95% significance. Power regression was used to identify a dose-dependent relationship between the amplified intensity and tested S-Np concentration. Clinical performance analysis of the AP-ART was determined via receiver operator characteristic (ROC) analysis. RT-PCR results were used to classify participants to positive/negative cases and cycle threshold was calculated as a mean of the genes tested. AP-ART test line intensity cut-off of above 0.56 arbitrary units (a.u.) was used to objectively classify the results as positive and equal/below 0.56 a.u. as negative. Sensitivity and specificity were then determined at 95% confidence intervals using this cut-off value with RT-PCR results as reference. Positive and negative results in the C-ARTs were determined based on the manufacturer’s instructions. All statistical analyses were performed using GraphPad Prism (GraphPad software, San Diego, CA).

## Results

### AP-ART development and concept

**Fig. 1:**
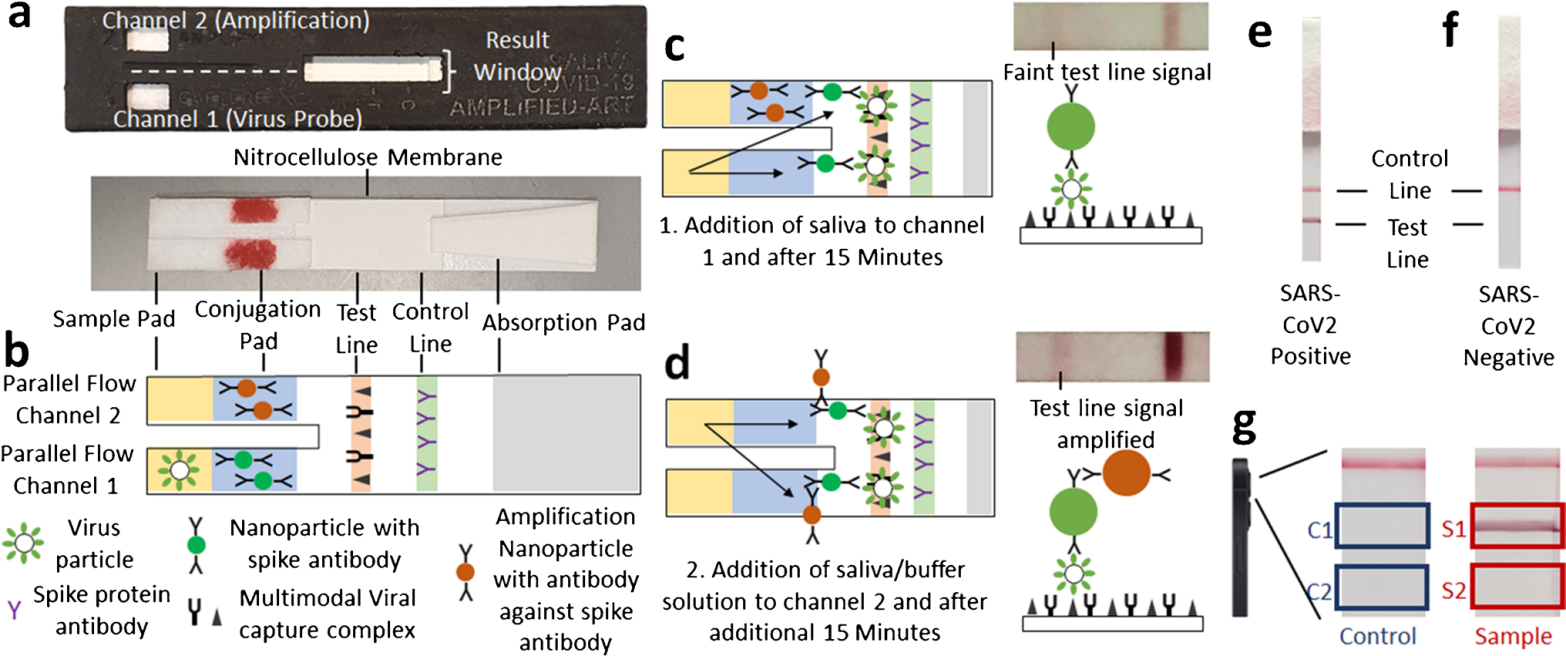
Method and working principle of the amplified parallel antigen rapid test (AP-ART). **a** Prototype showing two separate channels, **b** schematic diagram with multimodal viral capture complex at the test line, **c** sandwich assay showing virus isolated at test line tagged by nanoparticle with spike antibody after addition of saliva to channel 1, inset showing faint test line signal, **d** sandwich assay showing signal enhancement by amplification nanoparticle after addition of second saliva sample/buffer solution, inset showing increased test line intensity, **e** positive result where both control and test lines are visible, **f** negative result where only the control line is visible and **g** objective recording of results using mobile phone photography and image processing

The AP-ART prototype is shown in Fig. 1a and its schematic drawing is represented in Fig. 1b. Each channel contained a well for sample loading. The parallel channels were linked to the same nitrocellulose membrane. Saliva loaded onto channel 1 would flow via capillary action and react with gold nanoparticles with spike antibodies in the channel. The resultant immune complex would then continue flowing until captured by the recombinant human ACE2 protein and polyclonal spike antibodies embedded at the test line after 15 min (Fig. 1c). The viral spike protein was thus captured at the test line by two molecules (Fig. S3a) instead of one (Fig. S3b). The same saliva sample was then loaded onto channel 2 to mobilise the signal amplifying gold nanoparticles (Fig. 1d). The amplifying nanoparticles contained antibodies which would bind to spike antibodies (Fig. S4a). Thus, the amplifying nanoparticles would bind directly to the nanoparticles with spike antibodies from channel 1. This binding was achieved without the use of any linker molecule (Fig. S4b). This reaction formed a nanoparticle complex (Fig. S4c) that amplified the test line signal. A positive test would show visible test and control lines (Fig. 1e); a negative test would only produce a visible control line (Fig. 1f). Test and control lines were also captured using a mobile phone camera for objective measurements via image processing algorithms. Test line intensity was obtained by subtracting the mean intensity of Area B2 from the mean intensity of Area B1. Similarly, the control line intensity was obtained by subtracting the mean intensity of Area A2 from the mean intensity of Area A1 (Fig. 1g).

### Nanoparticle characterisation

**Fig. 2:**
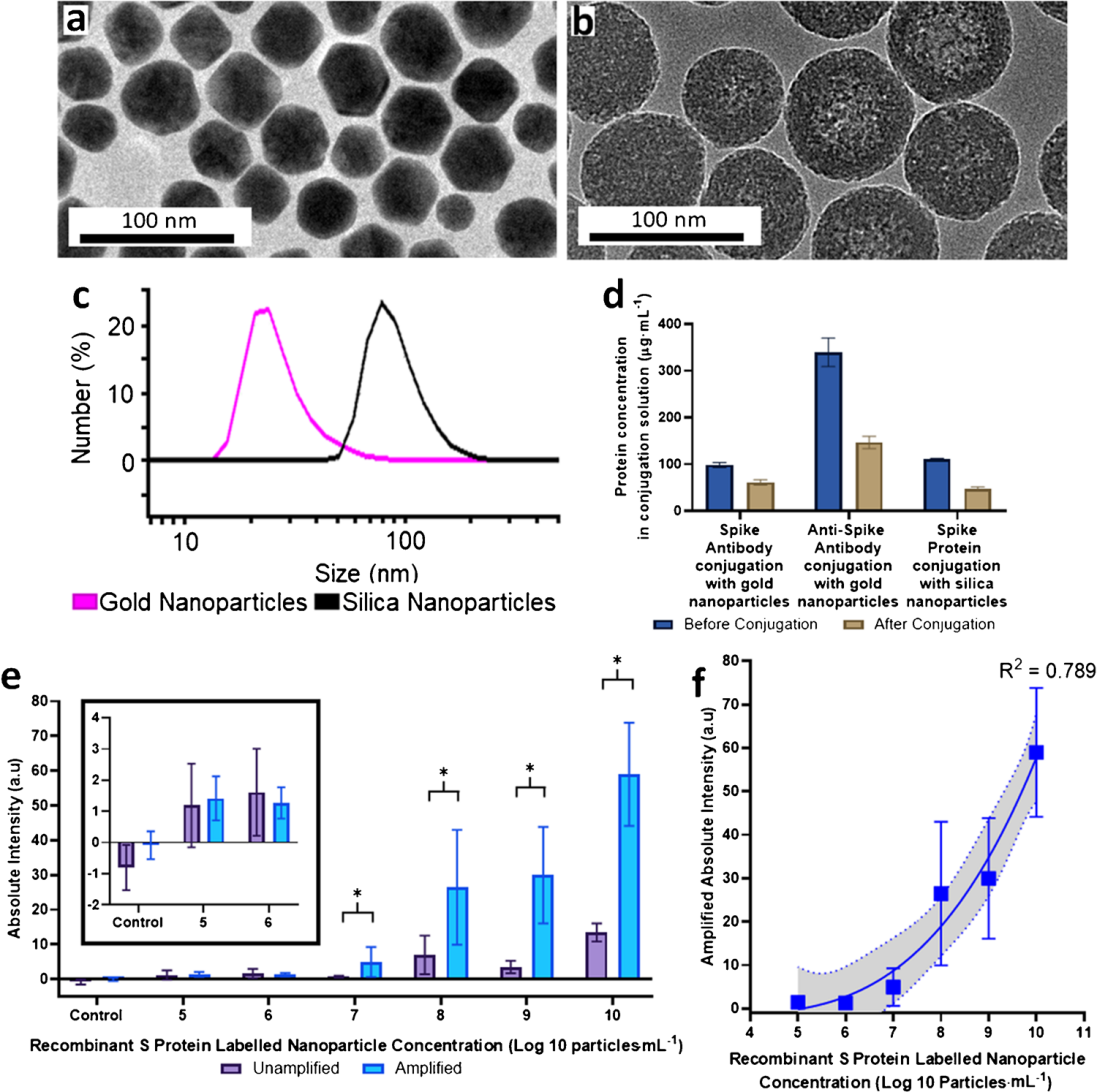
Nanoparticle characterisation and performance of AP-ART. **a** Transmission electron microscopy (TEM) of unconjugated gold nanoparticles, **b** TEM of unconjugated silica nanoparticles, **c** size distribution of conjugated gold and silica nanoparticles, **d** protein and antibody conjugation assay showing the protein concentration in the conjugation solution before and after conjugation for 4 h to illustrate the amount of protein bound to the respective nanoparticles (*N* = 3, concentration before conjugation has been adjusted for handling loses using a control experiment), **e** test line absolute intensity when unamplified and after amplification after testing with different concentrations of labelled particles, inset shows zoomed in view when testing with control, 10^5^ and 10^6^ particles⋅mL^−1^ (*N* = 5 repeats for each labelled particle concentration, all points were statistically significant from control at 95% confidence interval using *T* test. *Statistically significant at 95% confidence interval using *T* test) and **f** power series analysis showing dose response relationship of the amplified line intensities based on tested labelled particle concentration with 95% confidence interval

The structure of the unconjugated gold nanoparticles was imaged using transmission electron microscopy (TEM) as in Fig. 2a, showing a spherical shape and sizes with a mean diameter of 37.5 ± 5.88 nm. Similarly, unconjugated silica nanoparticles were imaged using TEM in Fig. 2b, showing a spherical morphology with a diameter of 75.5 ± 6.84 nm. This was close to the mean size of the SARS-CoV-2 virion (70 ± 6 nm) without its spike proteins as seen in TEM studies [24]. The silica nanoparticle size was chosen to serve as a positive control for the AP-ART assay after surface conjugation of spike proteins. Furthermore, being colourless, silica would not interfere with the colorimetric interpretation of the assay. The gold and silica nanoparticles after conjugation with the respective antibodies or proteins had a peak diameter of 44.1 ± 10.7 nm and 92.3 ± 27.4 nm, respectively, as seen in Fig. 2c. Two sets of gold nanoparticles were conjugated. One set was conjugated with SARS-CoV-2 spike protein binding antibodies. Another set functioned as amplification nanoparticles which contained secondary antibodies which bound to the spike protein binding antibodies from the first set, thus amplifying the signal. Silica nanoparticles were conjugated with SARS-CoV-2 Spike proteins. The protein and antibody conjugation to each nanoparticle is illustrated in Fig. 2d, which shows the concentration of the respective protein or antibody to be conjugated, before and after the conjugation. The measurements were corrected for handling losses by performing a control experiment using no nanoparticles. It was calculated that 102 spike antibody molecules were conjugated per analyte binding gold nanoparticle, 503 secondary antibodies were conjugated per amplification gold nanoparticle and 378 spike protein molecules were conjugated per silica nanoparticle. The detailed calculation is provided in Table S2 of the supplement.

### *In vitro *assessment of sensitivity

To demonstrate the detection range of AP-ART, we first applied an in vitro approach by measuring test line absolute intensity against different concentrations of recombinant S protein-labelled nanoparticles, as shown in Fig. 2e. This approach enabled us to overcome the need to conduct our preclinical studies in a high biological containment laboratory. For consistency, this in vitro assessment was performed using the same batch of nanoparticle conjugates to avoid inter-batch variation in S protein conjugation onto the nanoparticles. Test line intensities before and after activation of channel 2 for amplification were captured. Activation of channel 2 significantly increased the test band intensity as compared to without amplification (Fig. 2f). A detectable signal was observed at the test line when tested with S protein-labelled nanoparticles at the concentration of 10^5^ particles⋅mL^−1^, which can be calculated as 0.0064 ng⋅mL^−1^ of spike protein, based on the protein binding assay performed earlier. We also observed a dose-dependent relationship between the S protein-labelled nanoparticle concentration and the absolute intensity of the test line (power series fit *R*^2^ = 0.789). The gold nanoparticles produced a strong colorimetric signal when tested with the S protein-labelled nanoparticles. These characterisation results show that gold was an ideal nanomaterial due to its colorimetric properties and ease of conjugation with antibodies [25]. The temporal stability of the gold nanoparticles in the test kit was further evaluated as illustrated in Fig. S5 of the supplement. The ability of the AP-ART to detect the S protein-labelled nanoparticles was observed to be similar to fresh kits after 6 months of storage in a dry environment.

### Prospective clinical evaluation of AP-ART

**Fig. 3:**
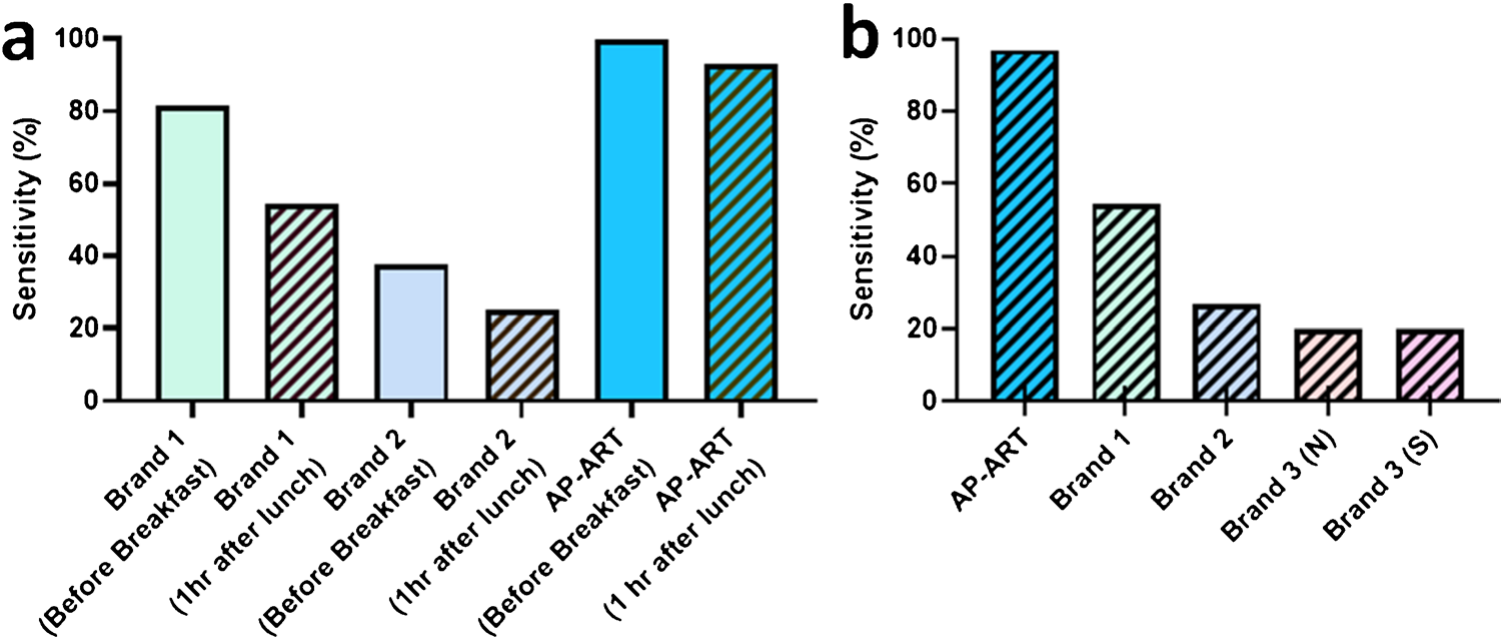
Time point testing showing the effect of oral intake on test sensitivity of the AP-ART compared with conventional antigen rapid tests (C-ART) from various brands in RT-PCR positive participants. **a** Sensitivity using overnight saliva (before breakfast) versus saliva after oral intake (1 h after lunch) in the same 11 patients in the AP-ART and C-ART from 2 brands* and **b** sensitivity of AP-ART compared to C-ARTs from various brands using saliva after oral intake (1 h after lunch).^■^ *A total of 11 participants were tested for brand 1 and AP-ART but only 8 participants were used for brand 2. ^■^The numbers of participants tested with each brand were AP-ART, 33; brand 1, 27; brand 2, 10; and brand 3 (N) and brand 3 (S), same group of 9 participants

A major limitation for any saliva-based diagnostic is the timing of sampling [26, 27]. Previous studies have shown that the sensitivity of saliva-based diagnostics is optimal before teeth-brushing or breakfast in the morning [16]. To validate this, 2 commercially available conventional ARTs (C-ARTs) were used to test saliva from 11 participants at two time points, before breakfast (with overnight fasting) and 1 h after lunch (Fig. 3a). For brand 1, sensitivity before breakfast was 81.8%, but after lunch, it was reduced to 54.5%. A similar reduction in sensitivity was noted for brand 2, when sensitivity before breakfast was 35.5%, but 1 h after lunch was only 25%. In contrast, the sensitivity of AP-ART was comparable before breakfast (100%) compared to 1 h after lunch (93.3%) (Fig. 3a). We further tested AP-ART against these 2 brands and another 2 C-ARTs from brand 3 using after 1-h post-lunch saliva from an additional group of participants (16 additional participants with C-ARTs and 22 additional participants with AP-ART), brands 1, 2 and 3 (N) test for the nucleocapsid protein and brand 3 (S) tests for the spike protein. The sensitivity of AP-ART was 97.0% and those of the other C-ARTs ranged from 20 to 54.6%. Both brand 3 (N) and brand 3 (S) had a sensitivity of 20% in the same group of participants (Fig. 3b). These results collectively suggest that the signal amplification step in AP-ART was able to overcome the post-meal dilution effect that negatively impacts C-ARTs.

We next conducted a case–control study to assess the performance of AP-ART using saliva samples obtained 1 h after lunch. A total of 33 COVID-19 cases and 106 controls (85 healthy HCWs and 19 patients with non-COVID-19 ARI) were included. Saliva samples from the cases were obtained within 72 h of a RT-PCR test on nasopharyngeal swab samples for SARS-CoV-2. The cases and controls tested positive and negative, respectively, by RT-PCR. Of the cases, 15 (45.5%) were infected with the delta variant of SARS-CoV-2, as verified by sequencing. A total of 56 saliva samples were collected via longitudinal sampling from RT-PCR positive patients whereas only a single sample was obtained from each of the RT-PCR negative controls. Thus, a total of 162 saliva samples were available for analysis (Fig. S2).

**Fig. 4:**
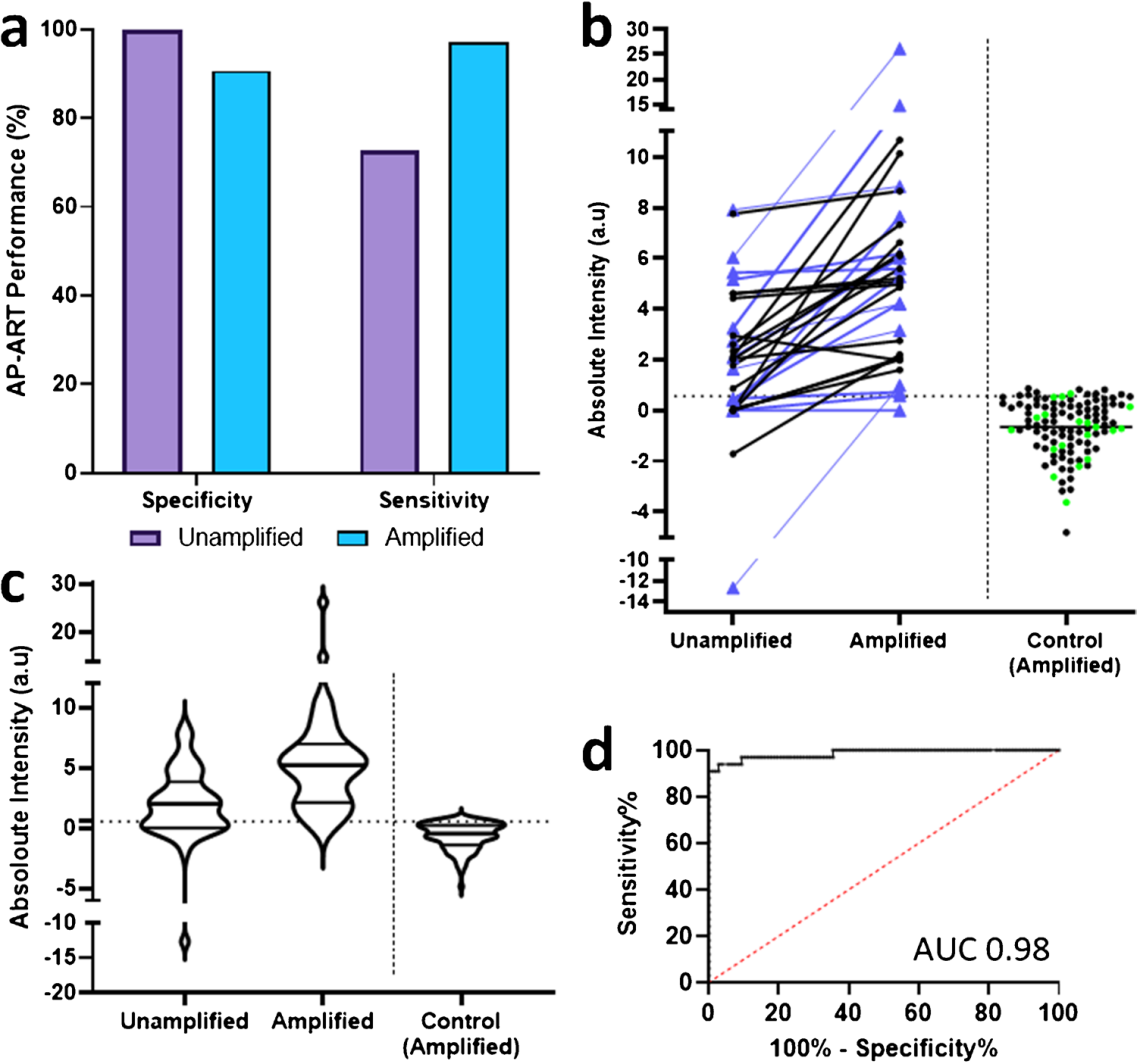
Clinical performance of AP-ART. **a** Clinical sensitivity and specificity of the AP-ART when unamplified and after amplification, **b** changes in each participant’s AP-ART test line absolute intensity before amplification and after amplification, sequencing data for 15 participants were available and these participants were found to have the delta variants (labelled with coloured ▲ symbols), 18 non-COVID-19 ARI participants were included in the control and were highlighted in green, **c** violin plot showing absolute test line intensity of AP-ART before and after amplification (horizontal line indicates the line intensity of 0.56 a.u. which is the threshold condition for a positive test), **d** receiver operator characteristic curve of AP-ART compared against PCR as the gold standard with area under the curve (AUC) of 0.98. Total number of PCR positive participants was 33 and PCR negative participants were 106 for all 4 figures

The clinical sensitivity of the AP-ART was 97.0% (95% CI: 84.7–99.8) after amplification, which was markedly better than 72.7% (95% CI: 83.7–94.8) without amplification (Fig. 4a). With amplification, 8 cases that were negative before amplification became positive. One case remained negative despite amplification (Fig. 4b). Amongst the 106 who were SARS-CoV-2 RT-PCR negative, all but 10 tested as negative by AP-ART. The control cases had an average amplified signal intensity of − 0.65 a.u, thus indicating that amplification did not cause any intensity increase in control samples (Fig. 4c). The test specificity was thus 90.6% (95% CI: 83.7–94.8). The cross tabulation of the AP-ART against the RT-PCR is provided in Table S3 of the supplement. In this study, 18 ARI participants were included in the control. Seventeen of 18 of these participants tested negative with the AP-ART and 5 of these participants had been tested positive for other pathogens such as *Klebsiella pneumoniae*, Rhinovirus, *Legionella*, dengue and group G streptococcus. One of the participants tested falsely positive with the AP-ART also tested positive for *Klebsiella pneumoniae*. A summary of the clinical diagnoses for the ARI participants is included in Table S4. For sensitivity analysis, a cut-off identity of 0.56 a.u for test positivity was obtained from a receiver operator characteristic (ROC) analysis using RT-PCR as reference (Fig. 4d). An area under the curve (AUC) of 0.98 (95% CI: 0.96–1.00) was obtained, showing good agreement with RT-PCR. Additionally, to determine if the AP-ART was affected by the vaccination status of the participants, an additional 10 fully vaccinated participants who had a positive RT-PCR for SARS-CoV-2 were tested. All participants tested positive after amplification and the results are shown in Fig. S6.

**Fig. 5:**
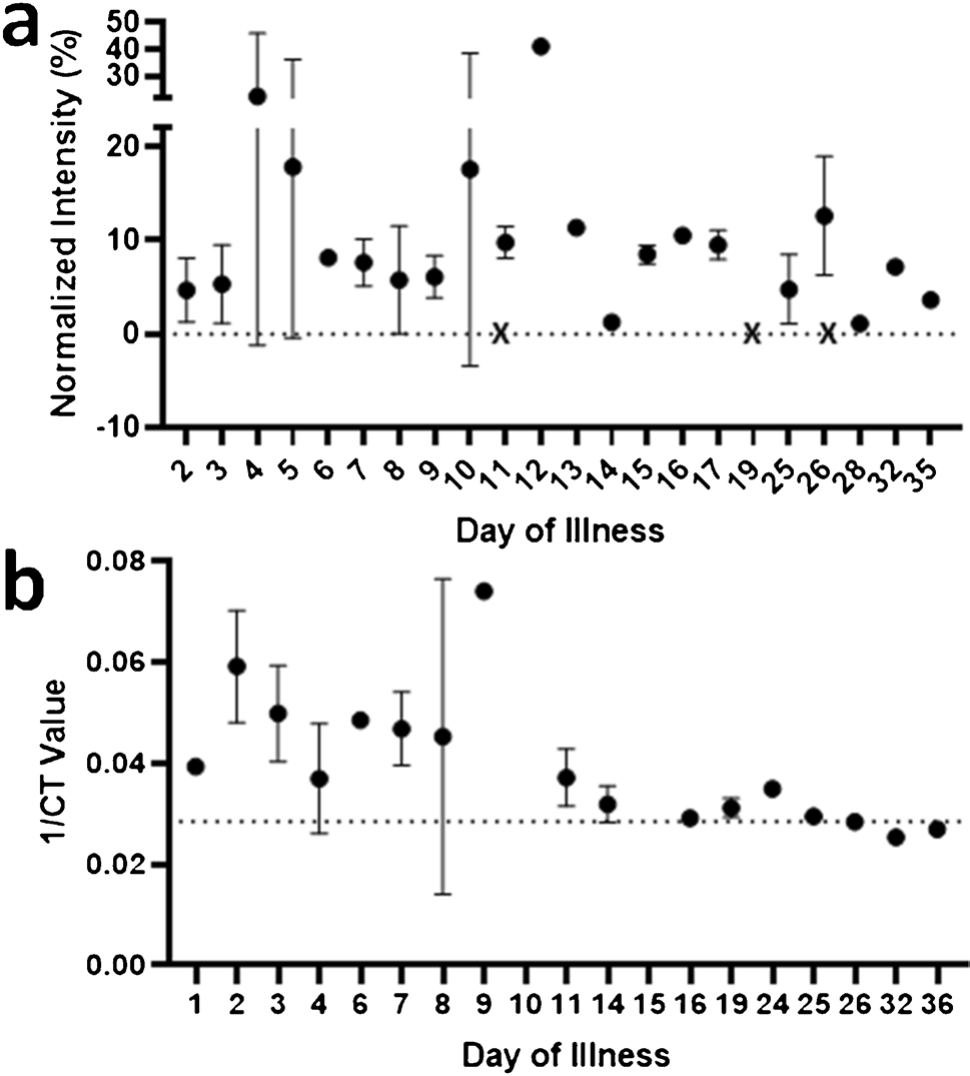
Longitudinal performance and comparison against PCR testing. **a** Mean normalised intensity of test line according to day of illness of patients tested serially on consecutive days, horizontal line at 0 is the threshold where > 0 indicates a detectable signal at the test line, and **b** mean reciprocal CT values of patients tested serially according to day of illness, horizontal line at *y* = 0.028 is the CT value of 35 which corresponds to the deisolation criteria for COVID-19-infected patients in Singapore.^§^ Crosses indicate results which did not show a detectable signal at the test line and were excluded from the means analyses. Total number of saliva samples analysed from the cohort of 40 patients was 56. ^§^Total number of PCR samples analysed from the cohort of 33 patients was 46

Finally, to define the illness period suitable for applying AP-ART, we compared AP-ART against RT-PCR in the 33 RT-PCR positive participants using longitudinal sampling until discharge (Fig. 5). A total of 58 saliva samples were obtained from 33 COVID-19 patients. Similarly, saliva samples were collected within 72 h of the nasopharyngeal swabs. Positive signals on AP-ART could be detected up to day 35 post-illness onset as compared to 25 days using RT-PCR, suggesting that the window for case detection could be longer than RT-PCR. Additionally, throughout the period when AP-ART remained positive, an average normalised line intensity of 10.1% compared to the control line was observed. Taken together, these results suggest that AP-ART was able to show a similar positive signal regardless of the day of illness when the test was performed.

## Discussion

The management of COVID-19 is constantly evolving. Early diagnosis is still a challenge, especially in healthcare resource-limited towns and cities in LMICs. Moreover, the successful development of efficacious antiviral drugs against SARS-CoV-2 infection further underscores the need for point-of-care tests for early diagnosis. Clinical trials of such drugs have shown maximal efficacy if these drugs were administered as early as possible after illness onset; delayed treatment diminishes the clinical benefit of treatment. Though there have been many commercially developed tests so far, the common underlying limitation of these tests are that they either require trained professionals for sample collection or laboratory support for sample processing. These commercially available methods are summarised in Table S5 of the supplement. Therefore, RT-PCR has remained the gold standard, and there is a global need for a high-sensitivity test which can produce similar results as RT-PCR at the point-of-care.

In this study, it was demonstrated that a signal amplified ART using saliva samples can approach the clinical sensitivity of RT-PCR in detecting SARS-CoV-2 infection in nasopharyngeal swabs. The inclusion of a second lateral flow channel allowed the introduction of linker-free nanoparticles that would react with those in complex with the SARS-CoV-2 S protein in the saliva, and thus amplify the test line, without reducing the reaction time with the analyte. In the presented approach, linker-free amplification nanoparticles that directly bind to the spike protein binding nanoparticles from the first channel were used. This simplifies the antibody conjugation process, and conserves binding spots on the nanoparticle surface for spike antibodies, as no linker is needed, in contrast to conventional dual gold amplification techniques. In the in vitro characterisation, AP-ART demonstration detection of up to 0.0064 ng⋅mL^−1^, compared to 0.01 ng⋅mL^−1^, is observed in conventional dual gold amplification methods. Though there are other methods for signal enhancement, most of these methods either do not offer as great as a degree of signal enhancement, have a poorer limit of detection or cannot be performed at the point-of-care. These methods and their limitations are summarised in Table S6 of the supplement. Additionally, though the test lines of the AP-ART can be interpreted by eye, the incorporation of a simple image processing algorithm, which can be made available in any mobile phone [28], could also be used to provide semi-quantitative results (as we have used in this study). This algorithm compared the intensity of the test and control line with that of the background to objectively determine the test line presence and quantitate its intensity. Our study also showed the ability of AP-ART to detect variants, and indeed sequencing data has shown it has the capability to detect the delta variants. Lastly, one important design consideration was the inclusion of ACE2 protein for functional capture at the test line. Theoretically, the AP-ART could become even more specific, as the S protein evolves to increase binding affinity with its cognate entry receptor [29]. The study data also suggests that the AP-ART is reasonably selective in its detection of SARS-CoV-2 with only one patient tested falsely positive out of 18 non-COVID-19 ARI patients.

The study of saliva instead of nasal swabs in this study was chosen for several reasons. Firstly, tests that rely on nasal swabs are operator-dependent [30], and thus, a saliva-based test circumvents this issue and can be easily self-administered [31]. Secondly, several studies have found that saliva may in fact harbour a higher viral load than nasopharyngeal secretions [32]. However, a key limitation of saliva samples is that it is readily diluted after food or water ingestion that limits diagnostic test performance [33], especially for ARTs [17, 18]. The requirement for fasting saliva sample for diagnosis would curtail the deployment of any diagnostic test, particularly in primary healthcare setting. The amplification step in AP-ART was able to overcome this dilutional effect for sustained high level sensitivity, and thus underscores the potential of AP-ART to be applied for point-of-care diagnosis. Thirdly, SARS-CoV-2 spike antigens have also been observed to persist longer than the genetic material tested in RT-PCR, which may be due to the difference in the circulating half-life these markers as seen in other viral studies [34]. Another postulation for the antigenic persistence could be due to residual depots of viral antigens which persist in the body in areas such as draining lymph nodes seen in other studies involving other respiratory pathogens [35]. For example, in animal models infected with the respiratory syncytial virus, the viral antigen can remain detectable for at least 5 weeks despite viral clearance as correlated with serological testing [36]. Fourthly, AP-ART successfully detected SARS-CoV-2 in the saliva even in patients whose nasopharyngeal swab samples had converted from RT-PCR to negative. Fifthly, the turnaround time of 30 min of AP-ART is an added advantage compared to RT-PCR. Finally, though there are no amplified ARTs similar to the AP-ART on the market, as the material cost of similar ARTs can be as low as USD 0.50 [37], it is expected that the AP-ART would also be highly affordable.

## Limitations

There are several limitations to the presented study. AP-ART remains a laboratory prototype and could be further optimised to reduce the time needed to complete the test. The study was also conducted as a case–control study design to evaluate the performance of AP-ART against RT-PCR. A prospective study would still be needed to determine its true performance relative to RT-PCR. Furthermore, though our control population correctly tested as negative except for one participant, further cross-reactivity studies of the AP-ART against other common viruses and especially human coronaviruses would be advantageous. In this study, the temporal stability of the test kit evaluated at 6 months showed that it has similar analytical sensitivity to detect the S protein to the concentration of 10^7^ particles⋅mL^−1^. However, as seen in Fig. S5, the intensity of these test lines was weaker than freshly made kits. Refinement of packaging and storage conditions may improve the long-term stability of this test kit. Of note, there have been other studies which showed stability of up to 5 years with minimal impact on performance [38]. Finally, although the study was able to analyse the test line intensity using mobile phone photography for objective recording, comparisons of the test intensities in participants may be difficult, as the lighting conditions varied between different isolation rooms. Future studies could also take this into account image acquisition design to obtain uniform lighting.

## Conclusion

In this clinical study, the proposed AP-ART demonstrated a sensitivity of 97%, approaching that of the gold standard RT-PCR testing in the detection of SARS-CoV-2. This was achieved though multimodal viral capture and signal enhancement using linker-free, signal amplification gold nanoparticles introduced via a parallel flow channel. This approach has overcome the limitations in previous dual gold amplified ART assays to achieve a one order of magnitude improvement in the limit of detection. This performance has allowed the AP-ART sensitivity to maintain its sensitivity even when using non-fasting saliva, unlike conventional ARTs. Furthermore, longitudinal saliva testing also demonstrated that in COVID-19 patients, viral antigens persisted longer than molecular markers, underscoring the potential of such high-sensitivity ARTs for ruling out infection. Overall, these results highlight the potential of the AP-ART as an easy-to-use point-of-care diagnostic test for early COVID-19 diagnosis.

## Supplementary Information

Below is the link to the electronic supplementary material.

Supplementary file1 (DOCX 1.28 MB)

## Data Availability

All clinical data was obtained from the Singapore General Hospital patient database and was de-identified at point of data processing. The dataset will be made available to academic parties on request from the corresponding investigator in accordance with data sharing policies of Singhealth (SG) and Duke-NUS Medical School (SG), with input from the investigator of the group where applicable.
