# Correction to: Amplified parallel antigen rapid test for point-of-care salivary detection of SARS-CoV-2 with improved sensitivity

**DOI:** 10.1007/s00604-022-05625-7

**Published:** 2023-01-07

**Authors:** Danny Jian Hang Tng, Bryan Chu Yang Yin, Jing Cao, Kwan Ki Karrie Ko, Kenneth Choon Meng Goh, Delia Xue Wen Chua, Yong Zhang, Melvin Lee Kiang Chua, Jenny Guek Hong Low, Eng Eong Ooi, Khee Chee Soo

**Affiliations:** 1 Department of Infectious Diseases, Singapore General Hospital, 20 College Road, Singapore, 169856 Singapore; 2 Programme in Emerging Infectious Diseases, Duke-NUS Medical School, 8 College Road, Singapore, 169857 Singapore; 3 Department of Head and Neck and Thoracic Cancers, Division of Radiation Oncology, National Cancer Centre Singapore, 11 Hospital Crescent, Singapore, 169610 Singapore; 4 Department of Biomedical Engineering, National University Singapore, 4 Engineering Drive 3, Engineering Block 4, Singapore, 117583 Singapore; 5 State Key Laboratory for Oncogenes and Related Genes, School of Biomedical Engineering and Institute of Medical Robotics, Shanghai Jiao Tong University, Shanghai, 200030 People’s Republic of China; 6 Department of Microbiology, Singapore General Hospital, 20 College Road, Singapore, 169856 Singapore; 7 Division of Medical Sciences, National Cancer Centre Singapore, 11 Hospital Crescent, Singapore, 169610 Singapore; 8 Oncology Academic Programme, Duke-NUS Medical School, 8 College Road, 169857 Singapore, Singapore

**Correction to: Microchimica Acta (2021) 189:14**

**https://doi.org/10.1007/s00604-021-05113-4**

In this article, there is an error in the concentration of Ab01680 antibodies used. It should be 1000 μg⋅mL^− 1^ instead of 100 μg⋅mL^− 1^. The corrected line should read as: "Briefly, using the lateral flow reagent dispenser, control lines were drawn using 200 μg⋅mL^− 1^ of mouse IgG and test lines were drawn using a solution consisting of Ab01680 antibodies (1000 μg⋅mL^− 1^) and ACE2 protein (100 μg⋅mL^− 1^) at a rate of 0.125 mL⋅min^− 1^ on nitrocellulose membrane".

The original article has been corrected.

