# Supplementary material for: Amplified parallel antigen rapid test for point-of-care salivary detection of SARS-CoV-2 with improved sensitivity
Source: Mikrochim Acta. 2021 Dec 6;189(1):14. doi: 10.1007/s00604-021-05113-4 (PMC8646336; doi:10.1007/s00604-021-05113-4)
Supplement: Supplementary file 1 — Supplementary file1 (DOCX 1.28 MB) [file 604_2021_5113_MOESM1_ESM.docx]

Electronic Supporting Material

**Amplified parallel antigen rapid test for point-of-care salivary detection of SARS-CoV-2 with improved sensitivity**

**Danny Jian Hang Tng,^1,2,3,*^ Bryan Chu Yang Yin,^2^ Jing Cao,^4,5^Kwan Ki Karrie Ko,^6^ Kenneth Choon Meng Goh,^6^ Delia Xue Wen Chua,^4^ Yong Zhang,^4^Melvin Lee Kiang Chua,^3,7,8^ Jenny Guek Hong Low,^1,2^ Eng Eong Ooi,^2^ Khee Chee Soo^7,8,*^**

*Co-corresponding authors

Affiliations:

^1^Department of Infectious Diseases, Singapore General Hospital, 20 College Road, Singapore, 169856, Singapore

^2^Programme in Emerging Infectious Diseases, Duke-NUS Medical School, 8 College Rd, Singapore, 169857, Singapore

^3^Department of Head and Neck and Thoracic Cancers, Division of Radiation Oncology, National Cancer Centre Singapore, 11 Hospital Crescent, Singapore, 169610, Singapore

^4^Department of Biomedical engineering, National University Singapore, 4 Engineering Drive 3, Engineering Block 4, Singapore, 117583, Singapore

^5^State Key Laboratory for Oncogenes and Related Genes, School of Biomedical Engineering and Institute of Medical Robotics, Shanghai Jiao Tong University, Shanghai, 200030, People’s Republic of China

^6^Department of Microbiology, Singapore General Hospital, 20 College Road, Singapore, 169856, Singapore

^7^Division of Medical Sciences, National Cancer Centre Singapore, 11 Hospital Crescent, Singapore, 169610, Singapore

^8^Oncology Academic Programme, Duke-NUS Medical School, 8 College Rd, Singapore, 169857, Singapore

| Reagent | Catalog Number | Manufacturer |
| --- | --- | --- |
| 40 nm Gold nanoparticles, Carboxylic Acid Functional Groups, Polyethylene Glycol, Molecular Weight 3000, OD 50 in water | [GFC-3K-40](https://www.cd-bioparticles.com/p/Carboxyl-Gold-Nanoparticles_258_259_269_2811.html) | Creative Diagnostics |
| 100 nm Silica Nanoparticles, Carboxylic Acid Functionalized | [DNG-F050](https://www.cd-bioparticles.com/p/DiagNano™-Carboxyl-Silica-Nanoparticles-100-nm_258_262_291_3767.html) |  |
| N-(3-Dimethylaminopropyl)-N-ethylcarbodiimide hydrochloride (EDC) | [E1769](https://www.sigmaaldrich.com/SG/en/product/sial/e1769?context=product) | Sigma Aldrich |
| N-Hydroxysulfosuccinimide sodium salt (NHS) | [56485](https://www.sigmaaldrich.com/SG/en/product/aldrich/56485?context=product) |  |
| 4-Morpholineethanesulfonic acid,  2-(N-Morpholino)ethanesulfonic acid hydrate (MES) | [M8250](https://www.sigmaaldrich.com/SG/en/product/sigma/m8250?context=product) |  |
| Horse Serum Antigen (HSA) | [008-000-121](https://www.jacksonimmuno.com/catalog/products/008-000-121) | Jackson Immunosciences |
| Human Angiostensin Converting Enzyme 2 protein (ACE2) | [abx652520](https://www.abbexa.com/human-angiotensin-i-converting-enzyme-2-ace2-protein) | Abbexa |
| Goat anti-mouse IgG H&L Alexa Fluor® 647 | [ab150115](https://www.abcam.com/goat-mouse-igg-hl-alexa-fluor-647-ab150115.html) | Abcam |
| Human origin Anti-COVID-19 / SARS-CoV S glycoprotein antibody | [Ab01680](https://absoluteantibody.com/product/anti-covid-19-sars-cov-s-glycoprotein-cr3022/) | Absolute antibody |
| Mouse origin SARS-CoV-2 (2019-nCoV) spike S1 antibody | [40591-MM42](https://www.sinobiological.com/antibodies/cov-spike-40591-mm42) | Sinobiological |
| SARS-CoV-2 (2019-nCoV) Spike RBD-mFc recombinant protein | [40592-V05H](https://www.sinobiological.com/recombinant-proteins/2019-ncov-cov-spike-40592-v05h) |  |
| Nitrocellulose membrane FF170HP Plus | [10547043](https://www.cytivalifesciences.com/en/us/shop/protein-analysis/immunoassays-biochemical-assays-accessories-and-reagents/components-for-lateral-flow-and-flow-through-immunoassays/ffhp-plus-nitrocellulose-membranes-p-10020) | Whatman |
| Absorption Pads CF5 | [8115-2250](https://www.cytivalifesciences.com/en/us/shop/whatman-laboratory-filtration/whatman-dx-components/lateral-flow-pads/cf5-p-00708) |  |
| Conjugation Pad Standard 14 | [8133-2250](https://www.cytivalifesciences.com/en/us/shop/whatman-laboratory-filtration/whatman-dx-components/lateral-flow-pads/standard-14-p-00709) |  |
| Backing Card | [MIBA-050](https://dcndx.com/store/product/lateral-flow-backing-cards/) | DCN Diagnostics |
| Automated lateral flow reagent dispenser (ALFRD) | [ALFRD](https://www.claremontbio.com/Lateral-Flow-Reagent-Dispenser-p/07.711.01.htm) | ClaremontBio |
| Centrifuge | [5424R](https://online-shop.eppendorf.com.my/MY-en/Centrifugation-44533/Microcentrifuges-1007183/Centrifuge-5425-5425R-PF-934144.html#epService) | Eppendorf |
| Thermomixer | [Comfort](https://sg.vwr.com/store/product/596278/thermomixer-thermomixer-comfort) |  |
| Particle Sizer/ NanoSight | [LM10](https://www.malvernpanalytical.com/en/products/product-range/nanosight-range/nanosight-lm10/) | Malvern Analytical |
| Dynamic Light Scattering | [Zetasizer Nano](https://www.malvernpanalytical.com/en/support/product-support/zetasizer-range/zetasizer-nano-range/zetasizer-nano-s) | Malvern Analytical |
| Spectrophotometer | [Spark 10M](https://lifesciences.tecan.com/multimode-plate-reader) | Tecan |
| Nanodrop | [Nanodrop 2000](https://www.thermofisher.com/order/catalog/product/ND2000CLAPTOP) | Thermofischer |

**Table S1.** Reagents and equipment

| Protein / Antibody | Nanoparticle | Mass of Protein/Antibody loss from solution after conjugation (µg⋅mL^-1^) | Molecular weight (KDa) | Molar concentration (nmol⋅mL^-1^) | No of protein/ antibody particles | No of protein/ antibody particles per nanoparticle |
| --- | --- | --- | --- | --- | --- | --- |
| Spike Antibody | Gold (1.5 x 10^12^ particles⋅mL^-1^) | 37.1 | 146 | 0.254 | 1.53 x 10^14^ | 102 |
| Anti-Spike Antibody | Gold (1.5 x 10^12^ particles⋅mL^-1^) | 193 | 156 | 1.25 | 7.55 x 10^14^ | 503 |
| Spike Protein | Silica (1.0 x 10^12^ particles⋅mL^-1^) | 63.6 | 102 | 0.627 | 3.78 x 10^14^ | 378 |

**Table S2.** Calculation of Protein or Antibody Binding per Gold or Silica Nanoparticle.


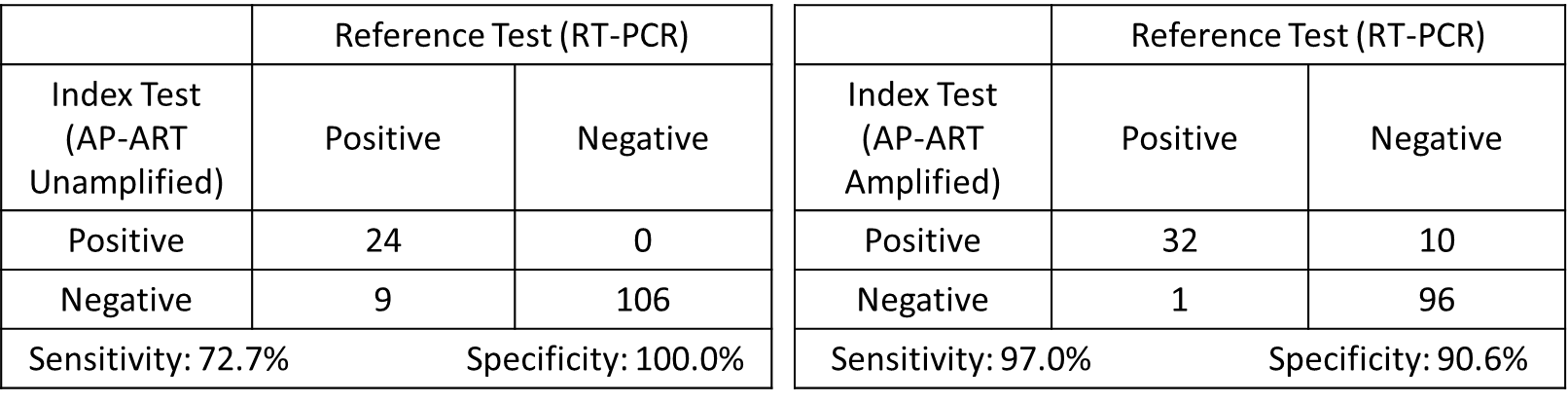


**Table S3.** Cross tabulation of the index test (AP-ART) results before and amplification compared to the reference test results (RT-PCR).

| **ID** | **Clinical Diagnosis** | **AP-ART** | **SARS-CoV-2 RT-PCR** |
| --- | --- | --- | --- |
| ARI-01 | Asthma exacerbation | Negative | Negative |
| ARI-02 | Asthma exacerbation | Negative | Negative |
| ARI-03 | Viral Upper Respiratory Tract Infection | Negative | Negative |
| ARI-04 | Klebsiella Pneumoniae | Negative | Negative |
| ARI-05 | Metapneumovirus (Rhinovirus) | Negative | Negative |
| ARI-06 | Rhinovirus ABC and Legionella Pneumonia | Negative | Negative |
| ARI-07 | Viral Lower Respiratory Tract Infection | Negative | Negative |
| ARI-08 | Dengue | Negative | Negative |
| ARI-09 | Viral Upper Respiratory Tract Infection | Negative | Negative |
| ARI-10 | Decompensated Congestive Cardiac Failure | Negative | Negative |
| ARI-11 | Group G streptococcus | Negative | Negative |
| ARI-12 | Viral Upper Respiratory Tract Infection | Negative | Negative |
| ARI-13 | Shortness of Breath and Vomiting in Pregnancy | Negative | Negative |
| ARI-14 | Viral Upper Respiratory Tract Infection | Negative | Negative |
| ARI-15 | Viral Upper Respiratory Tract Infection | Negative | Negative |
| ARI-16 | Viral Upper Respiratory Tract Infection | Negative | Negative |
| ARI-17 | Viral Upper Respiratory Tract Infection | Negative | Negative |
| ARI-18 | Interstitial Lung Disease Exacerbation | Negative | Negative |
| ARI-19 | Klebsiella pneumoniae | Positive | Negative |

**Table S4.** Clinical diagnoses of Patients from Acute Respiratory Illness Wards included in control population and corresponding test results using AP-ART and RT-PCR.

|  | **Amplified**  **antigen rapid testing**  **(This study)** | Conventional  Antigen rapid testing | Serological testing | Exhaled Breath Condensate “breathalyser” testing | Molecular Testing: Reverse Transcriptase - Polymerase Chain Reaction (RT-PCR) | Molecular Testing:  Reverse Transcription-Loop-Mediated isothermal Amplification (RT-LAMP) |
| --- | --- | --- | --- | --- | --- | --- |
| **Working Principle** | Detection of viral spike antigens | Detection of viral nucleocapsid antigens | Detection of specific antibodies (IgG, IgM, IgA) against viral antigen (spike, nucleocapsid, etc.) | Detection of volatile organic compounds [1] / genomic fragments associated with the virus [2] | Detection of viral genomic material | Detection of viral genomic material |
| **Main**  **Advantages** | - Can be performed at point-of-care | - Can be performed at point-of-care but results are operator dependent | - Can be performed at point-of-care | - Does not require highly skilled labour for sample collection | - Gold standard test when using nasal swabs | - Can be performed at point-of-care |
| **Main**  **Limitations** | - Not yet tested for other sample types | - If saliva samples are used: High rate of falsely negative results in unfasted patient  - If nasopharyngeal/ nasal samples are used: results are operator dependent [3] | - Trained staff needed to obtain blood samples  - Falsely positive in patients who are recently vaccinated or recovered from infection for up to 3 to 13 months [4-7] | - Requires laboratory support for sample processing and signal acquisition  - Patient must be well to perform coordinated breathing and provide sufficient samples [8] | - Requires trained staff for nasal swab collection  - Laboratory support needed for genomic extraction and amplification  - Saliva samples taken after meals have decreased viral load [9] | - Requires trained staff to perform amplification and sample collection  - Heating apparatus needed to maintain amplification temperature [10] |
| **Sample** | Saliva | Nasal Swab, Saliva, Oropharyngeal swab | Blood draw, capillary blood drop, saliva | Exhaled air | Nasal Swab, Saliva, Oropharyngeal swab | Nasal/Pharyngeal Swab |
| **Turnaround time** | 30 minutes | 15 - 30 minutes | 15 – 30 minutes | Few minutes (Volatile organic compounds)[1]  Few Hours (genomic fragments) [2] | 4 -6 hours | 20 minutes |
| **Sensitivity** | 97% | Nasopharyngeal [11-15]:  50 – 93.9%  Saliva [16, 17]:  11.7 – 23.1% | In unvaccinated patients with no recent infection:  Blood [18-23]:  IgG: 13 – 94%  IgA: 28 – 88%  IgM: 10 – 40%  Saliva [23]:  IgG: 13%  IgA: 28%  IgM: 10% | Detection of RNA fragments [8]:  68 – 93.5%  Volatile organic compounds [1]:  80 – 97.3 % | Nasopharyngeal [24, 25]:  84.8% - 92%  Saliva [24, 25]:  83.2 - 86% | 91% [10, 26] |
| **Specificity** | 90.6% | Nasopharyngeal [11-15]:  99.7 - 100%  Saliva [16, 17]:  100% | In unvaccinated patients with no recent infection:  Blood: [18-23]:  IgG: 96 - 100%  IgA: 98%  IgM: 95 - 100%  Saliva [23]:  IgG: 100%  IgA: 98%  IgM: 100% | Detection of RNA fragments [8]:  100%  Volatile organic compounds [1]:  100% | RT-PCR is the gold standard (100%) | 100% [10, 26] |

**Table S5.** Comparison of commercially available test kits for SARS-CoV-2.

| Signal Enhancement Type | Methodology | Main Limitations | Reported Limit of Detection | Detection Enhancement (number of folds of improvement) |
| --- | --- | --- | --- | --- |
| Direct test line signal enhancement | **Linker-free Dual gold enhancement with multimodal analyte capture. A second set of gold nanoparticles are used to complex with the first set of analyte binding nanoparticles without the use of linker molecules (This study).** | - Increases length of time of test (30 minutes vs 15 minutes of conventional test) | 0.0064 ng⋅mL^-1^  Or  0.0628 fmol | - |
|  | Dual gold enhancement. A second set of gold nanoparticles are used to complex with the first set of analyte binding nanoparticles [27, 28]. | - Requires linker molecules nanoparticle which will decrease the analyte binding capability of the analyte binding nanoparticles  - Amplification and analyte binding performed in the same channel reducing binding time | 0.01 - 0.06 ng⋅mL^-1^ | 30 - 100 |
|  | Silver lactate is applied at the test line to react with colloidal gold to produce an amplified colorimetric test line signal [29]. | - Some steps have to be performed in darkness  - Requires trained technician to perform amplification | 0.2 ng⋅mL^-1^ | 15 |
|  | Enzymatic reaction of Horseradish peroxidase complexed gold nanoparticles with Tetramethylbenzidine at the test line [30]. | - Requires trained technician to perform amplification | 0.2 ng⋅mL^-1^ | - |
|  | Polyamidoamine is used both as the analyte binding nanoparticle and also a center for gold aggregation [31, 32]. | - Similar limitations as dual gold enhancement | 0.1 ng⋅mL^-1^ | 20 - 50 |
|  | External thermal energy is used to excite metallic nanoparticles on the test line and subsequently imaged using a thermal sensor [33]. | - Additional equipment required to perform the thermal excitation and thermal imaging | - | 8 |
| Sample Concentration  (Indirect enhancement) | Magnetic analyte binding nanoparticles [34, 35]. | - External magnetic field is required to concentrate the bound nanoparticles  - Requires skilled technician to execute the multiple steps | 0.01 nM | 20 - 50 |
|  | Aqueous two-phase polymer-salt concentration system [36]. | - Long concentration time of 6 hours prevents rapid sample analysis  - Requires skilled technician to perform concentration and sample recovery | 1 × 10^9^ pfu⋅mL^-1^  (Plaque forming units per mL) | 10 |
|  | Paper-based sample concentration device using a semi-permeable membrane placed on top of PEGylated glass fiber [37]. | - Results differ based on concentration timing, user dependent results | 1.56  ng⋅mL^-1^ | 4 |
| Analyte binding nanoparticle modification  (Indirect enhancement) | Analyte binding fluorescent nanoparticles with oriented modified antibodies, based on specific binding of the Fc region of antibody with streptococcal protein G on the surface of polystyrene microspheres [38]. | - Requires fluorescence excitation and reader for result interpretation.  - Needs to be adapted for gold nanoparticle chemistries. | 0.032 ng⋅mL^-1^ | - |
|  | Increasing surface area of analyte binding nanoparticles by using gold nanopopcorns and gold nanostars instead of conventional gold spheres [39]. | - Few commercially sources of such hierarchical gold nanoparticles  - No long-term stability studies | 0.1 ng⋅mL^-1^ | 5 |
|  | Aminolated and Thiolated PEG-Covered Gold Nanoparticles with High Stability and Anti-aggregation [40]. | - Superior short-term stability versus conventional nanoparticles, few long-term stability studies | 0.472 ng⋅mL^-1^ | 12.5 |
|  | Gold nanoparticles conjugated with a nucleic acid probe [41]. | - Requires nucleic acid extraction and purification steps similar to PCR | 5 fmol | - |
|  | Core-shell gold-silica nanoparticles [42]. | - Superior short-term stability versus conventional nanoparticles, few long-term stability studies | 0.3 ng⋅mL^-1^ | 30 |
| Increasing nanoparticle binding time  (Indirect enhancement) | Hydrophobic wax-printed pillars to increase binding time between analyte and detection antibody [43]. | - Increases length of time of test  - Requires wax printing which may not be scalable for mass production | 6.5 ng⋅mL^-1^ | 3 |
|  | Additional membrane between conjugate and test pad for signal amplification by extending antigen/antibody binding interactions [44]. | - Increases length of time of test | 1 ng⋅mL^-1^ | 2 |

**Table S6.** Comparison of currently available approaches used to directly or indirect amplify test line signal.

**
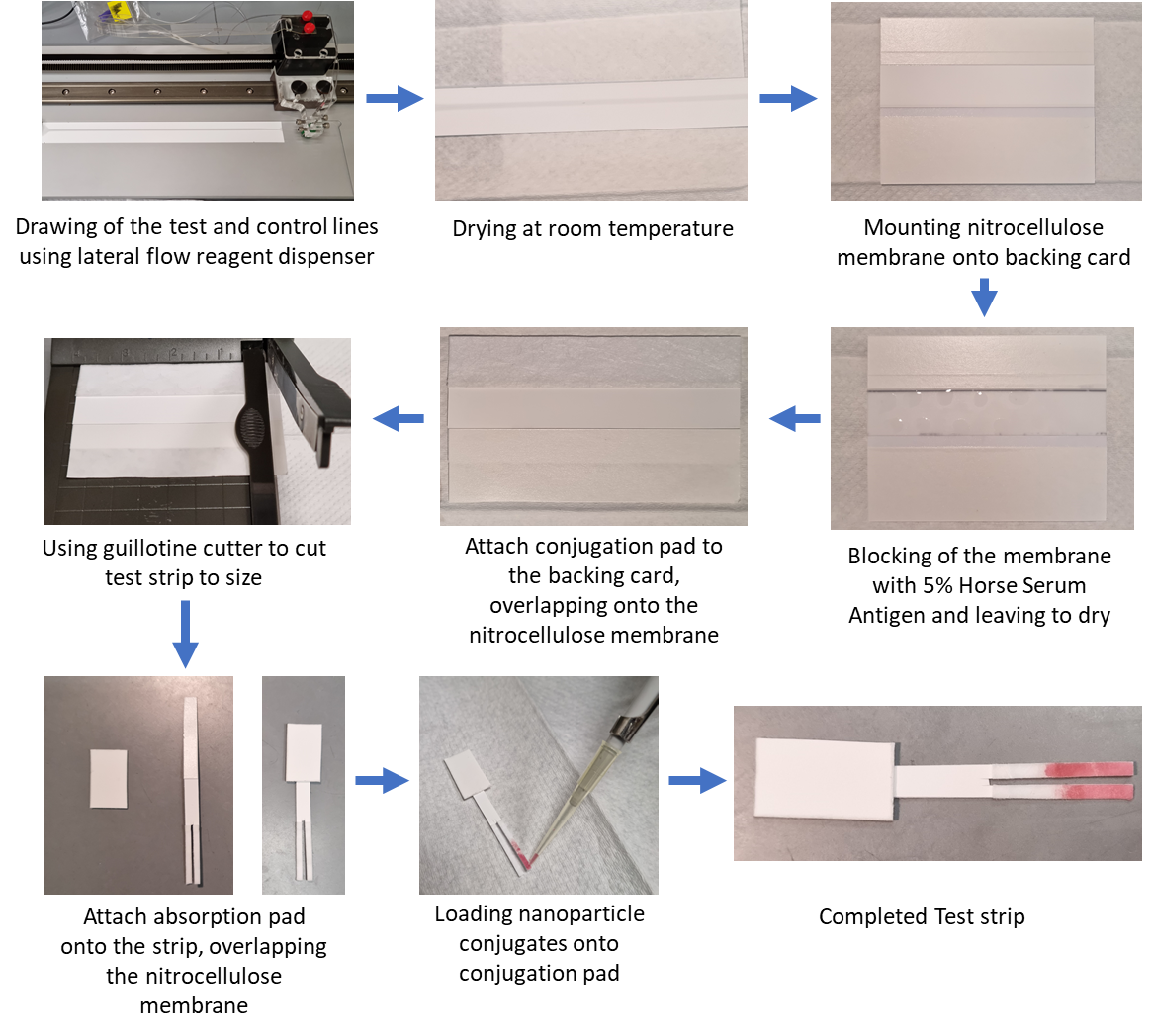
**

**Fig. S1.** Schema illustrating the preparation of the AP-ART.


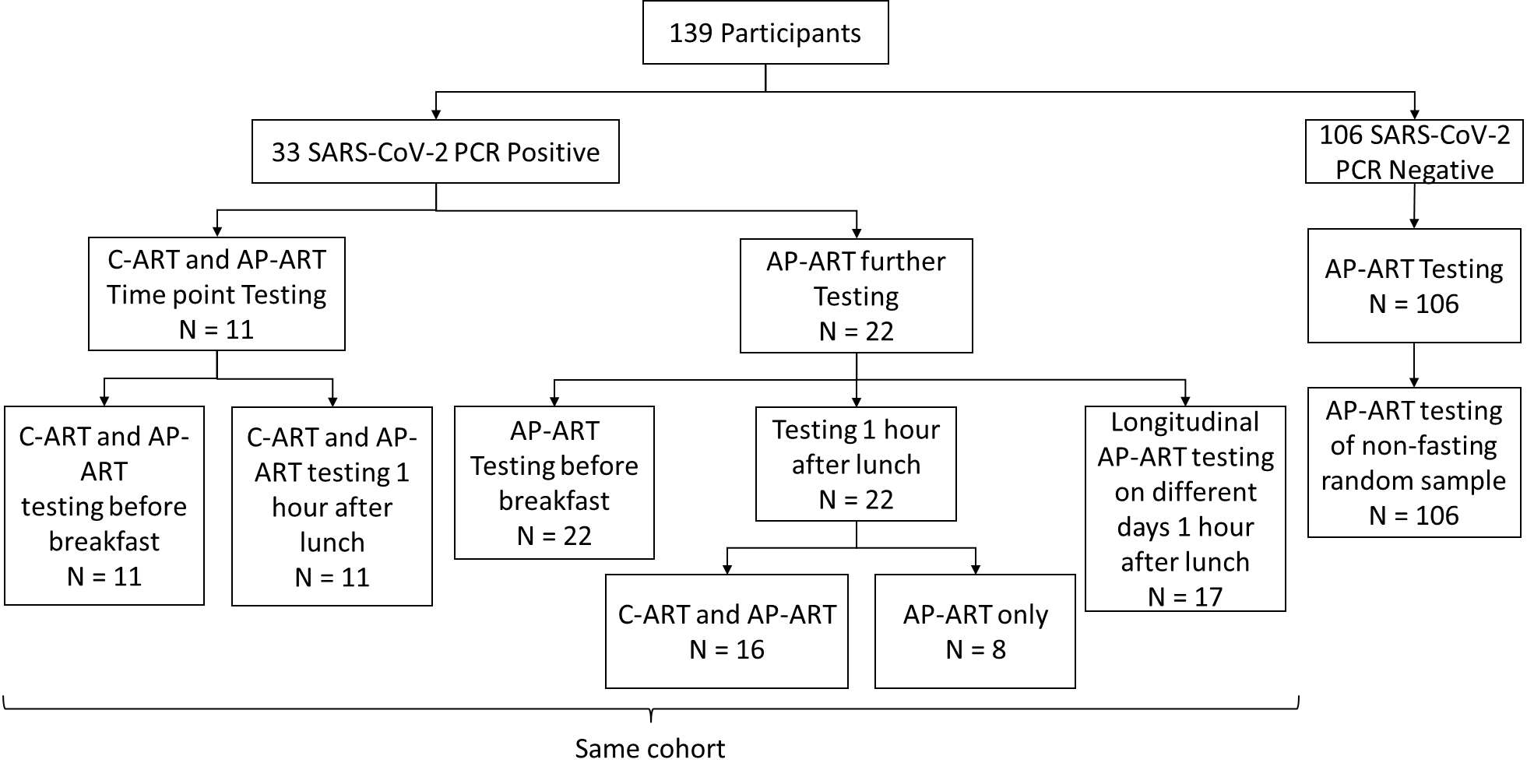


**Fig. S2**. Study design. Abbreviations: Conventional Antigen Rapid Test (C-ART) and Amplified Parallel flow Antigen Rapid Test (AP-ART). Saliva from the same cohort of 33 SARS-CoV-2 PCR positive participants were used to test the AP-ART and as well as C-ARTs from various brands in different situations. The cohort which was tested with both the C-ART and AP-ART was also included in the analysis.


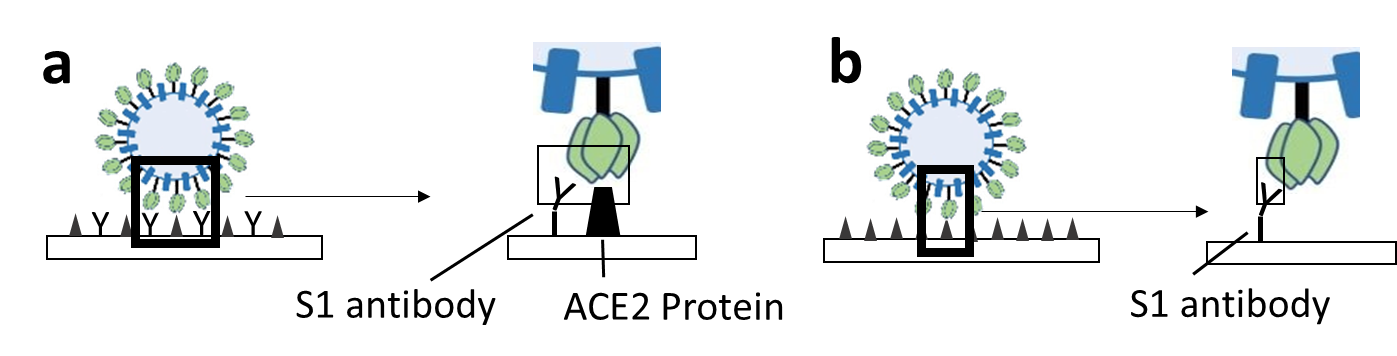


**Fig. S3**. Multimodal binding of spike protein at test line: (**a**) multimodal binding using ACE protein for functional binding of the virus to the RBD region and an S1 antibody for structural binding and (**b**) conventional virus capture using an antibody against the target antigen (for example S1 antibody in this case) showing binding mainly to the S1 region of the spike protein.

**
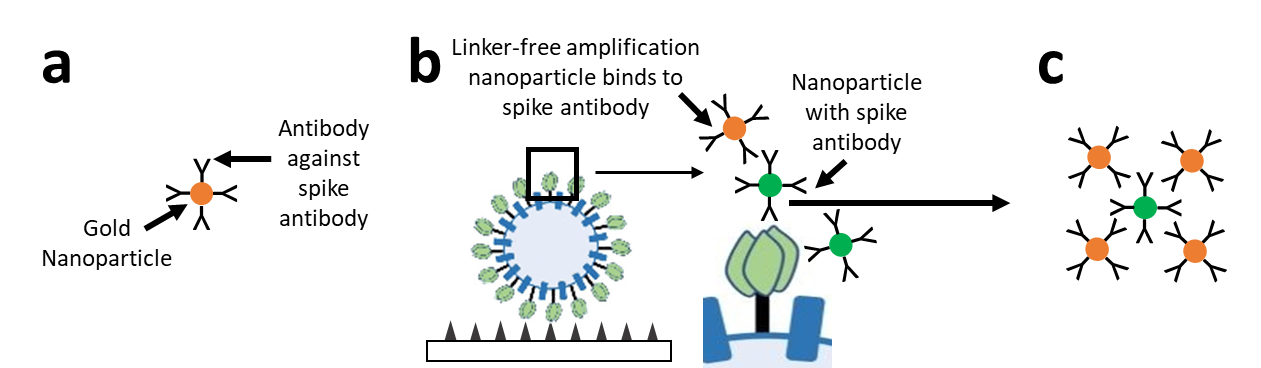
**

**Fig. S4**. Linker-free amplification approach amplification. (**a**) linker-free amplification particle with antibody against spike antibody, (**b**) linker-free amplification where amplification nanoparticle binds directly to spike antibodies, (**c**) expanded view of linker-free amplification nanoparticle complex.

**Fig. S5.** Temporal stability of AP-ART over time, comparing signal line intensity between kits which were stored in a dry cabinet for 6 months versus kits which were newly made. (Number of kits tested after storage for 6 months: 10^10^ particles⋅mL^-1^ – 2, 10^9^ particles⋅mL^-1^ – 2, 10^8^ particles/ml – 2, 10^7^ particles⋅mL^-1^ – 1 and control – 1)


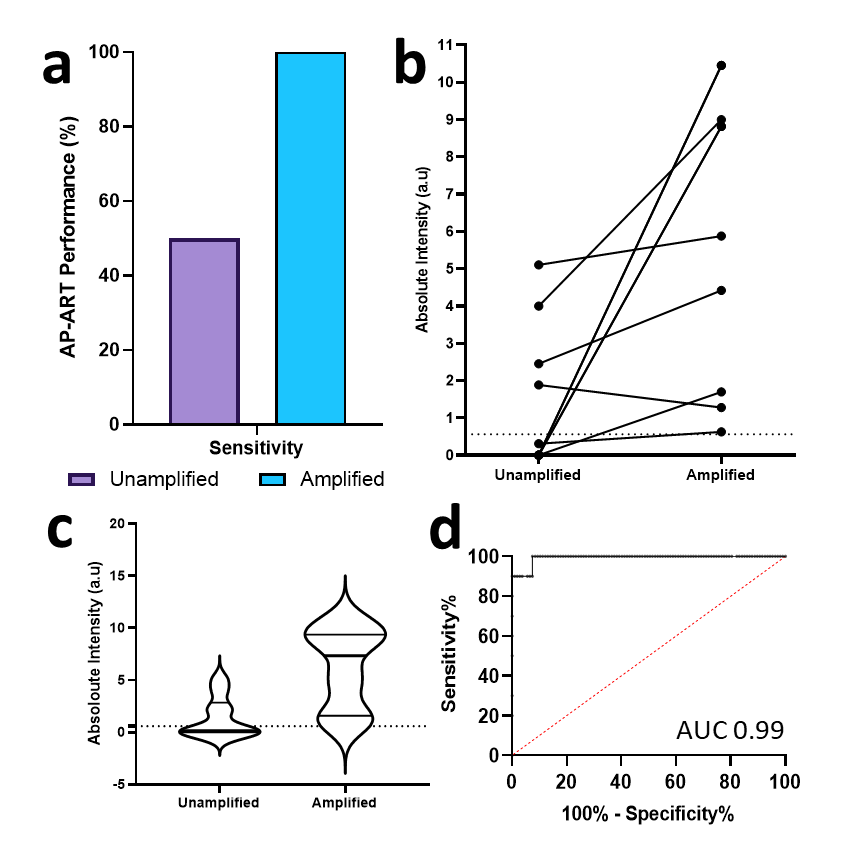


**Fig. S6.** Clinical performance of AP-ART in fully vaccinated participants. (**a**) Clinical sensitivity of the AP-ART when unamplified and after amplification, (**b**) changes in each participant’s AP-ART test line absolute intensity before amplification and after amplification, (**c**) violin plot showing absolute test line intensity of AP-ART before and after amplification (horizontal line indicates the line intensity of 0.56 a.u. which is the threshold condition for a positive test), (**d**) receiver operator characteristic (ROC) curve of AP-ART compared against PCR as the gold standard with Area Under the Curve (AUC) of 0.99. Total number of PCR Positive participants were 10 and the same 106 controls were used for ROC analysis.

**References**

[1] Steppert C, Steppert I, Sterlacci W, Bollinger T (2021) Rapid detection of SARS-CoV-2 infection by multicapillary column coupled ion mobility spectrometry (MCC-IMS) of breath. A proof of concept study. Journal of Breath Research.15(2):027105.https://doi.org/10.1088/1752-7163/abe5ca

[2] Chandrapalan S, Persaud K, Arasaradnam RP (2020) Breath diagnostics in the era of SARS-CoV-2—clinical and research arena. Journal of Breath Research.14(4):042002.https://doi.org/10.1088/1752-7163/ab924a

[3] Lindner AK, Nikolai O, Kausch F, Wintel M, Hommes F, Gertler M, Krüger LJ, et al. (2021) Head-to-head comparison of SARS-CoV-2 antigen-detecting rapid test with self-collected nasal swab versus professional-collected nasopharyngeal swab. European Respiratory Journal.57(4).https://doi.org/10.1183/13993003.03961-2020

[4] Isho B, Abe KT, Zuo M, Jamal AJ, Rathod B, Wang JH, Li Z, et al. (2020) Persistence of serum and saliva antibody responses to SARS-CoV-2 spike antigens in COVID-19 patients. Science immunology.5(52).https://doi.org/10.1126/sciimmunol.abe5511

[5] Gallais F, Gantner P, Bruel T, Velay A, Planas D, Wendling M-J, Bayer S, et al. (2021) Evolution of antibody responses up to 13 months after SARS-CoV-2 infection and risk of reinfection. EBioMedicine.71:103561.https://doi.org/10.1016/j.ebiom.2021.103561

[6] Levin EG, Lustig Y, Cohen C, Fluss R, Indenbaum V, Amit S, Doolman R, et al. (2021) Waning immune humoral response to BNT162b2 covid-19 vaccine over 6 months. New England Journal of Medicine.http://doi.org/10.1056/NEJMoa2114583

[7] Bayart J-L, Douxfils J, Gillot C, David C, Mullier F, Elsen M, Eucher C, et al. (2021) Waning of IgG, total and neutralizing antibodies 6 months post-vaccination with BNT162b2 in healthcare workers. Vaccines.9(10):1092.https://doi.org/10.3390/vaccines9101092

[8] Ryan DJ, Toomey S, Madden SF, Casey M, Breathnach OS, Morris PG, Grogan L, et al. (2021) Use of exhaled breath condensate (EBC) in the diagnosis of SARS-COV-2 (COVID-19). Thorax.76(1):86-8.http://dx.doi.org/10.1136/thoraxjnl-2020-215705

[9] Hung DL-L, Li X, Chiu KH-Y, Yip CC-Y, To KK-W, Chan JF-W, Sridhar S, et al. (2020) Early-morning vs spot posterior oropharyngeal saliva for diagnosis of SARS-CoV-2 infection: implication of timing of specimen collection for community-wide screening. Open forum infectious diseases: Oxford University Press US.https://doi.org/10.1093/ofid/ofaa210

[10] Mautner L, Baillie C-K, Herold HM, Volkwein W, Guertler P, Eberle U, Ackermann N, et al. (2020) Rapid point-of-care detection of SARS-CoV-2 using reverse transcription loop-mediated isothermal amplification (RT-LAMP). Virology journal.17(1):1-14.https://doi.org/10.1186/s12985-020-01435-6

[11] Lambert-Niclot S, Cuffel A, Le Pape S, Vauloup-Fellous C, Morand-Joubert L, Roque-Afonso A-M, Le Goff J, et al. (2020) Evaluation of a rapid diagnostic assay for detection of SARS-CoV-2 antigen in nasopharyngeal swabs. Journal of clinical microbiology.58(8):e00977-20.https://doi.org/10.1128/JCM.00977-20

[12] Porte L, Legarraga P, Vollrath V, Aguilera X, Munita JM, Araos R, Pizarro G, et al. (2020) Evaluation of a novel antigen-based rapid detection test for the diagnosis of SARS-CoV-2 in respiratory samples. International Journal of Infectious Diseases.99:328-33.https://doi.org/10.1016/j.ijid.2020.05.098

[13] Mahase E (2020) Covid-19: Innova lateral flow test is not fit for “test and release” strategy, say experts. BMJ: British Medical Journal (Online).371.https://doi.org/10.1136/bmj.m4469

[14] Holt E (2020) Slovakia to test all adults for SARS-CoV-2. The Lancet.396(10260):1386-7.https://doi.org/10.1016/S0140-6736(20)32261-3

[15] Cerutti F, Burdino E, Milia MG, Allice T, Gregori G, Bruzzone B, Ghisetti V (2020) Urgent need of rapid tests for SARS CoV-2 antigen detection: Evaluation of the SD-Biosensor antigen test for SARS-CoV-2. Journal of Clinical Virology.132:104654.https://doi.org/10.1016/j.jcv.2020.104654

[16] Agulló V, Fernández-González M, Ortiz de la Tabla V, Gonzalo-Jiménez N, García JA, Masiá M, Gutiérrez F (2021) Evaluation of the rapid antigen test Panbio COVID-19 in saliva and nasal swabs in a population-based point-of-care study. Journal of Infection.82(5):186-230.https://doi.org/10.1016/j.jinf.2020.12.007

[17] Nagura-Ikeda M, Imai K, Tabata S, Miyoshi K, Murahara N, Mizuno T, Horiuchi M, et al. (2020) Clinical Evaluation of Self-Collected Saliva by Quantitative Reverse Transcription-PCR (RT-qPCR), Direct RT-qPCR, Reverse Transcription–Loop-Mediated Isothermal Amplification, and a Rapid Antigen Test To Diagnose COVID-19. Journal of Clinical Microbiology.58(9).https://doi.org/10.1128/JCM.01438-20

[18] Iwasaki S, Fujisawa S, Nakakubo S, Kamada K, Yamashita Y, Fukumoto T, Sato K, et al. (2020) Comparison of SARS-CoV-2 detection in nasopharyngeal swab and saliva. Journal of Infection.81(2):e145-e7.https://10.1016/j.jinf.2020.05.071

[19] Ma H, Zeng W, He H, Zhao D, Jiang D, Zhou P, Cheng L, et al. (2020) Serum IgA, IgM, and IgG responses in COVID-19. Cellular & molecular immunology.17(7):773-5.https://doi.org/10.1038/s41423-020-0474-z

[20] Huang Z, Chen H, Xue M, Huang H, Zheng P, Luo W, Liang X, et al. (2020) Characteristics and roles of severe acute respiratory syndrome coronavirus 2‐specific antibodies in patients with different severities of coronavirus 19. Clinical & Experimental Immunology.202(2):210-9.https://doi.org/10.1111/cei.13500

[21] Van Elslande J, Houben E, Depypere M, Brackenier A, Desmet S, André E, Van Ranst M, et al. (2020) Diagnostic performance of seven rapid IgG/IgM antibody tests and the Euroimmun IgA/IgG ELISA in COVID-19 patients. Clinical Microbiology and Infection.26(8):1082-7.https://doi.org/10.1016/j.cmi.2020.05.023

[22] Lou B, Li T-D, Zheng S-F, Su Y-Y, Li Z-Y, Liu W, Yu F, et al. (2020) Serology characteristics of SARS-CoV-2 infection after exposure and post-symptom onset. European Respiratory Journal.56(2).https://doi.org/10.1183/13993003.00763-2020

[23] Yu H-q, Sun B-q, Fang Z-f, Zhao J-c, Liu X-y, Li Y-m, Sun X-z, et al. (2020) Distinct features of SARS-CoV-2-specific IgA response in COVID-19 patients. European Respiratory Journal.56(2).https://doi.org/10.1183/13993003.01526-2020

[24] Ibrahimi N, Delaunay-Moisan A, Hill C, Le Teuff G, Rupprecht J-F, Thuret J-Y, Chaltiel D, et al. (2021) Screening for SARS-CoV-2 by RT-PCR: Saliva or nasopharyngeal swab? Rapid review and meta-analysis. Plos one.16(6):e0253007.https://10.1371/journal.pone.0253007

[25] Butler-Laporte G, Lawandi A, Schiller I, Yao M, Dendukuri N, McDonald EG, Lee TC (2021) Comparison of saliva and nasopharyngeal swab nucleic acid amplification testing for detection of SARS-CoV-2: a systematic review and meta-analysis. JAMA Intern Med.181(3):353-8.https://doi.org/0.1001/jamainternmed.2020.8876

[26] Rodriguez-Manzano J, Malpartida-Cardenas K, Moser N, Pennisi I, Cavuto M, Miglietta L, Moniri A, et al. (2021) Handheld point-of-care system for rapid detection of SARS-CoV-2 extracted RNA in under 20 min. ACS central science.7(2):307-17.https://doi.org/10.1021/acscentsci.0c01288

[27] Shen Y, Shen G (2019) Signal-enhanced lateral flow immunoassay with dual gold nanoparticle conjugates for the detection of hepatitis B surface antigen. ACS Omega.4(3):5083-7.https://doi.org/10.1021/acsomega.8b03593

[28] Shen G, Zhang S, Hu X (2013) Signal enhancement in a lateral flow immunoassay based on dual gold nanoparticle conjugates. Clinical biochemistry.46(16-17):1734-8.https://doi.org/10.1016/j.clinbiochem.2013.08.010

[29] Panferov VG, Safenkova IV, Byzova NA, Varitsev YA, Zherdev AV, Dzantiev BB (2018) Silver-enhanced lateral flow immunoassay for highly-sensitive detection of potato leafroll virus. Food and Agricultural Immunology.29:445-57.https://doi.org/10.1080/09540105.2017.1401044

[30] Parolo C, de la Escosura-Muñiz A, Merkoçi A (2013) Enhanced lateral flow immunoassay using gold nanoparticles loaded with enzymes. Biosensors and Bioelectronics.40:412-6.https://doi.org/10.1016/j.bios.2012.06.049

[31] Peng X, Kang L, Pang F, Li H, Luo R, Luo X, Sun F (2018) A signal-enhanced lateral flow strip biosensor for ultrasensitive and on-site detection of bisphenol A. Food and Agricultural Immunology.29(1):216-27.https://doi.org/10.1021/acs.analchem.6b03406

[32] Shen G, Xu H, Gurung AS, Yang Y, Liu G (2013) Lateral flow immunoassay with the signal enhanced by gold nanoparticle aggregates based on polyamidoamine dendrimer. Analytical Sciences.29(8):799-804.https://doi.org/10.2116/analsci.29.799

[33] Wang Y, Qin Z, Boulware DR, Pritt BS, Sloan LM, González IJ, Bell D, et al. (2016) Thermal contrast amplification reader yielding 8-fold analytical improvement for disease detection with lateral flow assays. Analytical chemistry.88(23):11774-82.https://doi.org/10.1021/acs.analchem.6b03406

[34] Nash MA, Waitumbi JN, Hoffman AS, Yager P, Stayton PS (2012) Multiplexed enrichment and detection of malarial biomarkers using a stimuli-responsive iron oxide and gold nanoparticle reagent system. ACS nano.6(8):6776-85.https://doi.org/10.1021/nn3015008

[35] Ge X, Zhang W, Lin Y, Du D (2013) Magnetic Fe3O4@ TiO2 nanoparticles-based test strip immunosensing device for rapid detection of phosphorylated butyrylcholinesterase. Biosensors and Bioelectronics.50:486-91.https://doi.org/10.1016/j.bios.2013.07.017

[36] Jue E, Yamanishi CD, Chiu RYT, Wu BM, Kamei DT (2014) Using an aqueous two‐phase polymer‐salt system to rapidly concentrate viruses for improving the detection limit of the lateral‐flow immunoassay. Biotechnology and bioengineering.111(12):2499-507.https://doi.org/10.1002/bit.25316

[37] Tang R, Yang H, Choi JR, Gong Y, Hu J, Feng S, Pingguan-Murphy B, et al. (2016) Improved sensitivity of lateral flow assay using paper-based sample concentration technique. Talanta.152:269-76.https://doi.org/10.1016/j.talanta.2016.02.017

[38] Lou D, Fan L, Cui Y, Zhu Y, Gu N, Zhang Y (2018) Fluorescent nanoprobes with oriented modified antibodies to improve lateral flow immunoassay of cardiac troponin I. Analytical chemistry.90(11):6502-8.https://doi.org/10.1021/acs.analchem.7b05410

[39] Serebrennikova K, Samsonova J, Osipov A (2018) Hierarchical nanogold labels to improve the sensitivity of lateral flow immunoassay. Nano-micro letters.10(2):1-8.https://doi.org/10.1007/s40820-017-0180-2

[40] Lin LK, Uzunoglu A, Stanciu LA (2018) Aminolated and Thiolated PEG‐Covered Gold Nanoparticles with High Stability and Antiaggregation for Lateral Flow Detection of Bisphenol A. Small.14(10):1702828.https://doi.org/10.1002/smll.201702828

[41] Liu C-C, Yeung C-Y, Chen P-H, Yeh M-K, Hou S-Y (2013) Salmonella detection using 16S ribosomal DNA/RNA probe-gold nanoparticles and lateral flow immunoassay. Food chemistry.141(3):2526-32.https://doi.org/10.1016/j.foodchem.2013.05.089

[42] Lu X, Guo Q, Zhou W, Li X, Chen J, Zhou X, Sun N, et al. (2019) Improved performance of lateral flow immunoassays for alpha-fetoprotein and vanillin by using silica shell-stabilized gold nanoparticles. Microchimica Acta.186(1):1-7.https://doi.org/10.1007/s00604-018-3107-9

[43] Rivas L, Medina-Sánchez M, De La Escosura-Muñiz A, Merkoçi A (2014) Improving sensitivity of gold nanoparticle-based lateral flow assays by using wax-printed pillars as delay barriers of microfluidics. Lab on a Chip.14:4406-14.https://doi.org/10.1039/c4lc00972j

[44] Tsai T-T, Huang T-H, Chen C-A, Ho NY-J, Chou Y-J, Chen C-F (2018) Development a stacking pad design for enhancing the sensitivity of lateral flow immunoassay. Scientific reports.8:1-10.https://doi.org/10.1038/s41598-018-35694-9
